# Heartbeat OCE: corneal biomechanical response to simulated heartbeat pulsation measured by optical coherence elastography

**DOI:** 10.1117/1.JBO.25.5.055001

**Published:** 2020-05-05

**Authors:** Achuth Nair, Manmohan Singh, Salavat R. Aglyamov, Kirill V. Larin

**Affiliations:** a University of Houston, Department of Biomedical Engineering, Houston, Texas, United States; b University of Houston, Department of Mechanical Engineering, Houston, Texas, United States

**Keywords:** cornea, tissue biomechanics, pulsation, optical coherence elastography, optical coherence tomography

## Abstract

**Significance:** It is generally agreed that the corneal mechanical properties are strongly linked to many eye diseases and could be used to assess disease progression and response to therapies. Elastography is the most notable method of assessing corneal mechanical properties, but it generally requires some type of external excitation to induce a measurable displacement in the tissue.

**Aim:** We present Heartbeat Optical Coherence Elastography (Hb-OCE), a truly passive method that can measure the elasticity of the cornea based on intrinsic corneal displacements induced by the heartbeat.

**Approach:** Hb-OCE measurements were performed in untreated and UV-A/riboflavin cross-linked porcine corneas *ex vivo*, and a distinct difference in strain was detected. Furthermore, a partially cross-linked cornea was also assessed, and the treated and untreated areas were similarly distinguished.

**Results:** Our results suggest that Hb-OCE can spatially map displacements in the cornea induced by small fluctuations in intraocular pressure, similar to what is induced by the heartbeat.

**Conclusions:** The described technique opens the possibility for completely passive and noncontact *in vivo* assessment of corneal stiffness.

## 1 Introduction

Corneal pathologies such as corneal dystrophy, keratoconus, and corneal ectasia due to surgical complications can lead to corneal deformation and tissue damage, as well as underlying changes in intrinsic corneal biomechanical properties.^1^^,^^2^ Detecting the changes in these biomechanical properties is an emerging method for detecting ocular diseases, monitoring disease progression, and evaluating therapies because it is postulated that changes in tissue biomechanical properties can preclude structural or functional changes.^3^ Noncontact applanation tonometers such as the CorVis ST (OCULUS Optikgerate GmbH, Germany) and the ORA (Reichert Technologies, USA) have long been being used to measure corneal biomechanical properties by analyzing the response of the cornea to a large burst of air (inducing displacement of mm-scale). However, these methods have had conflicting results in measuring mechanical property changes because separating the effects from corneal geometry, biomechanical properties, and intraocular pressure (IOP) is a complex problem that is currently under investigation.^4^^–^^8^ Brillouin microscopy has also been effective in assessing the mechanical properties of the cornea indirectly,^9^^,^^10^ but spatial assessment of tissue elasticity may require long imaging times (∼50  s for a cross sectional scan^11^), and quantification of material parameters (e.g., Young’s modulus) from the Brillouin frequency shift is still an open question.^12^ In contrast, elastography has rapidly been growing since its inception in the 90s as a quantitative technique for measuring tissue biomechanical properties. In elastography, tissue motion is measured using an imaging modality,^13^^,^^14^ and then material parameters (e.g., Young’s modulus) are estimated by appropriate mechanical models. Ultrasound elastography and optical coherence elastography (OCE) have been previously utilized to assess the mechanical properties of the cornea.^8^^,^^15^^–^^18^ Typically, these techniques measure the corneal response to different external forces.^19^^–^^23^ However, there is growing interest in assessing the tissue mechanical response to intrinsic physiological forces.^24^ For example, hemodynamics within the eyeball can cause ocular tissue to experience pulsatile motion known as the ocular pulse. In other words, the ocular pulse is the difference between systolic and diastolic IOP. This pulse is highly correlated with biomechanical properties of the eye, and distinguishing changes in tissue during the various phases of the pulse may reveal important information about biomechanical properties of ocular tissues.^25^ Diffuse shear wave imaging that measured tissue response to muscular activity and the heartbeat has been demonstrated in an *in vivo* rat eye model.^26^ However, this noise correlation technique may not necessarily distinguish stiffness across different ocular tissues. Recently, ultrasound elastography has been used to measure the corneal biomechanical response to simulated quasi-static IOP fluctuations, but this method is limited by the resolution of its parent imaging modality.^27^^,^^28^ OCE has been utilized to measure the corneal response to slow diurnal fluctuations in IOP with a higher resolution. However, the ocular pulse and subsequent displacements in the cornea that occur at the heart rate are much faster than diurnal fluctuations and could be utilized to assess dynamic physiological pressures on the cornea.^29^^–^^31^ By taking advantage of the physiological forces applied to the corneal tissue, it is possible to assess the biomechanical properties of the cornea using only an OCT system, which is ubiquitous in ophthalmic clinics now. In this work, we introduce heartbeat OCE (Hb-OCE) to assess the corneal biomechanical response to varying fluctuations in IOP that simulate the heartbeat-induced ocular pulse in an *ex vivo* porcine cornea model. Furthermore, this technique was used to distinguish tissue stiffness between normal and crosslinked (CXL) corneas.

## 2 Materials and Methods

Three *ex vivo* porcine eye pairs were used for the pilot studies no more than 48 h after enucleation. Extraneous tissues, such as the eyelids and muscles, were removed, and each eye was placed into a custom eye holder in the whole eye-globe configuration. In each pair, one eye was used as an untreated (UT) control, while the fellow eye was crosslinked using the standard Dresden protocol.^32^ Corneas in the CXL group first had their epithelium removed using a microspatula. A solution of 0.1% riboflavin-5-phosphate in 20% Dextran T-500 was applied every 5 min on the cornea for a period of 30 min. Next, the cornea was irradiated with ultraviolet light (365 nm, 7 mm beam diameter, $3\;{\mathrm{mW}/\mathrm{cm}}^{2}$ intensity) for another 30 min while the riboflavin solution was applied every 5 min. The eye-globes were cannulated with two needles for IOP control using a home-built closed-loop IOP controller made up of a piezoresistive pressure transmitter (Model 41X, Keller AG für Druckmesstechnik, Switzerland) and syringe pump (NE-500, New Era Pump Systems Inc., USA). Briefly, a 1× phosphate-buffered saline (PBS) solution was infused into and withdrawn from the eye using the syringe pump. Fluid pressure (IOP) was measured through a needle inserted into the eye using the pressure transmitter. The IOP was precycled from 10 to 30 mmHg for four cycles prior to Hb-OCE measurement. The IOP was then set to a baseline pressure of 12 mmHg, which is within the normal IOP range.^33^ The IOP controller induced a sinusoidal pulsation with ∼1  mmHg amplitude and a highly repeatable 10 s period. Corneas were hydrated regularly at 2-min intervals using 1× PBS.

**Fig. 1:**
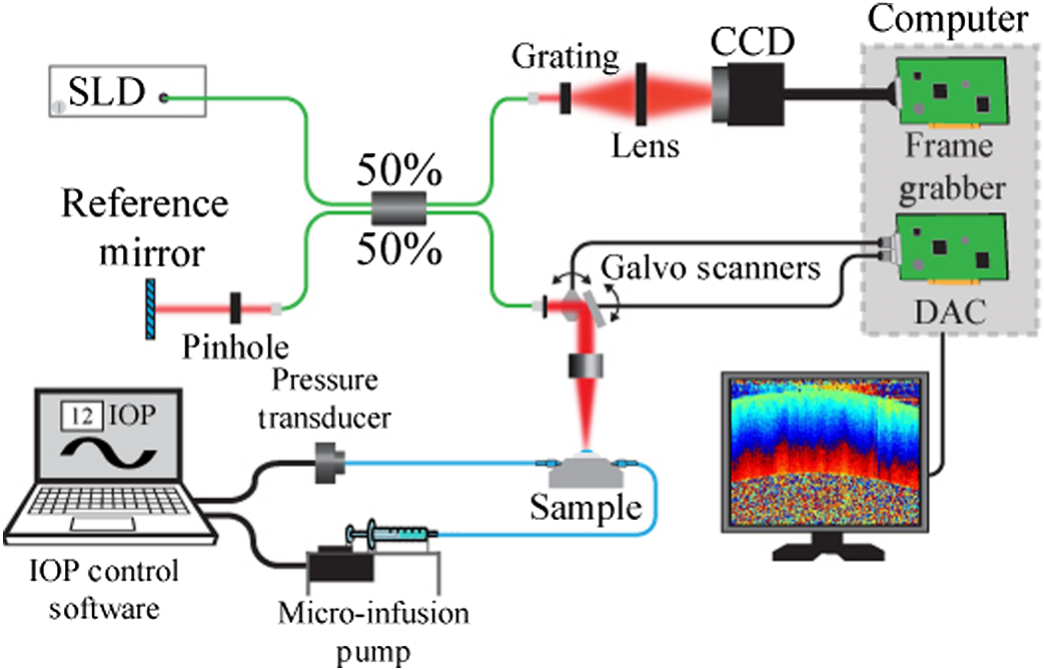
OCE system schematic. A closed-loop IOP controller-induced sinusoidal fluctuations in IOP. Induced displacements were measured with an SD-OCT system.

OCE data were acquired using a phase-sensitive spectral-domain optical coherence tomography system, which had an 840-nm central wavelength, 49-nm bandwidth, 6-μm axial resolution, 50-kHz line rate, and ∼5-nm nanometer displacement stability. B-mode images of 1000 A-lines across a 4-mm region were acquired at a 40-Hz frame rate. Figure 1 shows the OCE system schematic diagram.

An intensity-based thresholding method was used to segment each OCT B-scan and identify the top and bottom surfaces of the cornea. Motion detection was calculated based on the complex conjugate of consecutive OCT B-scans.^34^^,^^35^ The phase difference between pixels $\left(x,z,t_{0}\right)$ and $\left(x,z,t_{1}\right)$ in two consecutive frames was

$$\Delta \phi (x,z,t)=\text{angle}[I(x,z,t_{0})I^{*}(x,z,t_{1})],$$ (1)

where x is the lateral dimension, z is the depth dimension, I is the complex data t is the time, and * denotes the complex conjugate. To reduce noise in the phase difference image, vector averaging was performed with a 6×6  pixel window size.^36^ After two-dimensional-phase unwrapping,^37^ axial displacements $d_{\text{surface}}$ and $d_{\text{inside}}$ were obtained using the following equations on the surface of and inside the cornea, respectively:^17^^,^^38^

$$d_{\text{surface}}(z)=\Delta \phi_{\text{surface}}(z)\frac{\lambda_{0}}{4\pi n_{\text{air}}},$$ (2)

and

$$d_{\text{inside}}(z)=\frac{\lambda_{0}[\Delta \phi_{\text{inside}}(z)+\frac{\left(n_{\text{inside}}-n_{\text{air}}\right)}{n_{\text{air}}}\Delta \phi_{\text{surface}}(z)}{4\pi n_{\text{inside}}},$$ (3)

where $\lambda_{0}$ is the central wavelength of the OCT system and n=1.376 is the refractive index of the cornea.^39^^,^^40^ For each displacement frame, axial displacements were normalized to the corneal surface (i.e., all displacement for a given A-scan was subtracted from the displacement at the corneal surface at that A-scan). The average strain for each A-scan along the entire depth of the cornea at each point in time was calculated from the displacement using a least squares regression method.^41^ Briefly, the relationship between axial displacement and depth is determined within a sliding axial window. The strain value calculated within that window is determined by the slope of the linear regression with the least variance. The time lag between IOP fluctuation and displacement was compensated for in all strain figures. Untreated and CXL corneal pairs were compared based on the strain averaged across the entire cornea in the imaged region (∼4  mm) during a single simulated pulse period.

As a proof of concept for spatially resolved biomechanical mapping, a single porcine cornea that was partially crosslinked was also evaluated. Here, only half of the cornea underwent the CXL treatment, as described earlier. The untreated half was covered with aluminum foil to prevent riboflavin diffusion and UV irradiation. The same Hb-OCE measurements were performed as with the other corneas, with a 0.5 mmHg pulsation amplitude to reduce decorrelation artifacts.

## 3 Results

**Fig. 2:**
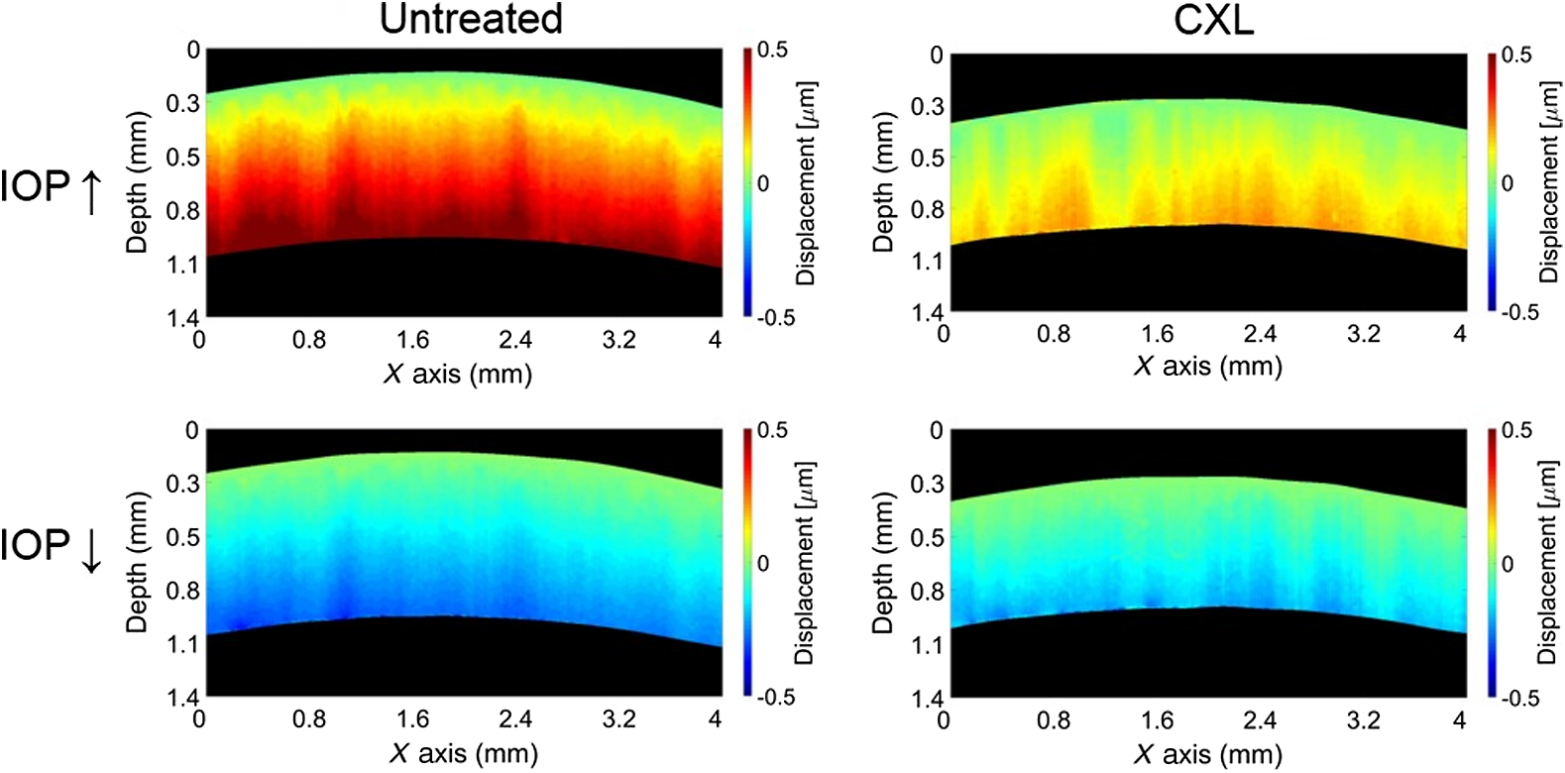
Corneal instantaneous displacement in the typical untreated and crosslinked corneas during infusion (IOP ↑, ∼2  s after the pulse starts) and withdrawal (IOP ↓, ∼6  s after the pulse starts) of the fluid from the eye-globe.

Our results demonstrate a cyclical corneal biomechanical response that corresponds to mechanical forces induced by fluid infusion into and withdrawal from the eye-globe due to the simulated IOP pulsation in both normal and CXL corneas. The instantaneous displacement maps during IOP increase (∼2  s into the pulse) and decrease (∼6  s into the pulse) in a representative sample are shown in Fig. 2. The measured displacement during IOP increase corresponds to the moment of liquid infusion into the eye-globe. Likewise, displacement measured during IOP decrease corresponds to liquid withdrawal from the eye-globe. After CXL, the thickness of the cornea noticeably reduces. The displacement in the untreated cornea has a significantly higher amplitude than the CXL cornea, suggesting a higher stiffness in the crosslinked tissue. Note that, since the corneal surface is used as a reference for normalization as described earlier, the displacement magnitude increases along the depth of the cornea from the surface. Variations in displacement across the cornea at particular depths are within a small percentage of the average displacement.

**Fig. 3:**
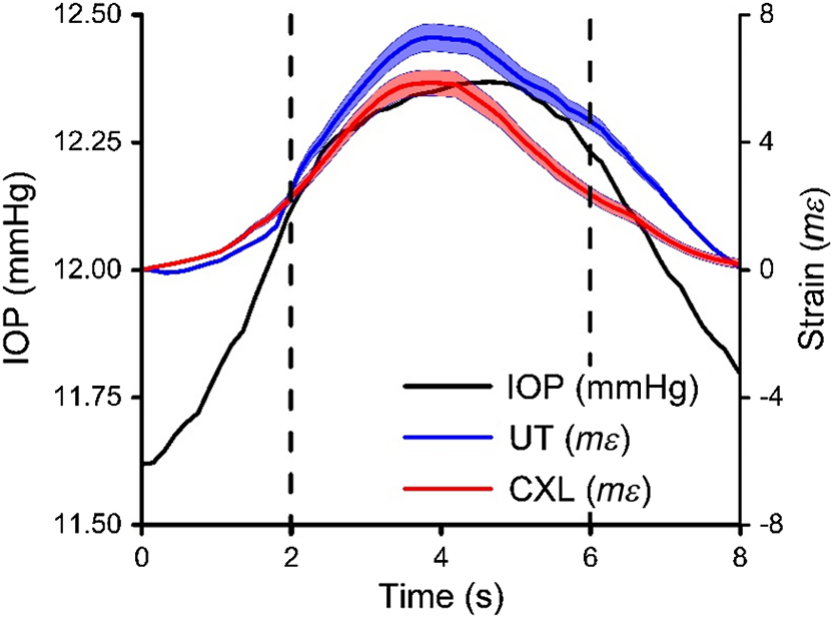
Cumulative strain for a typical untreated and CXL cornea. IOP over time is shown as well. Dotted lines represent points in time where corresponding displacement was mapped in Fig. 2.

The depth-averaged axial strain as calculated from the displacement at each time frame for a typical untreated and CXL cornea over an 8-s period is shown in Fig. 3. The IOP over time is also shown for comparison. Note the change in the cumulative strain over time. As IOP increased, strain increased as well, which is as expected. The magnitude of the change in strain in the untreated cornea is greater than the CXL cornea magnitude, indicating a greater stiffness in the CXL cornea. IOP shows a slightly faster infusion of the liquid into the eye than withdrawal, which results in the asymmetric fluctuation in strain as shown. In addition, the difference in width between the untreated and CXL strain curves may be attributed to increased stiffness due to crosslinking.

**Fig. 4:**
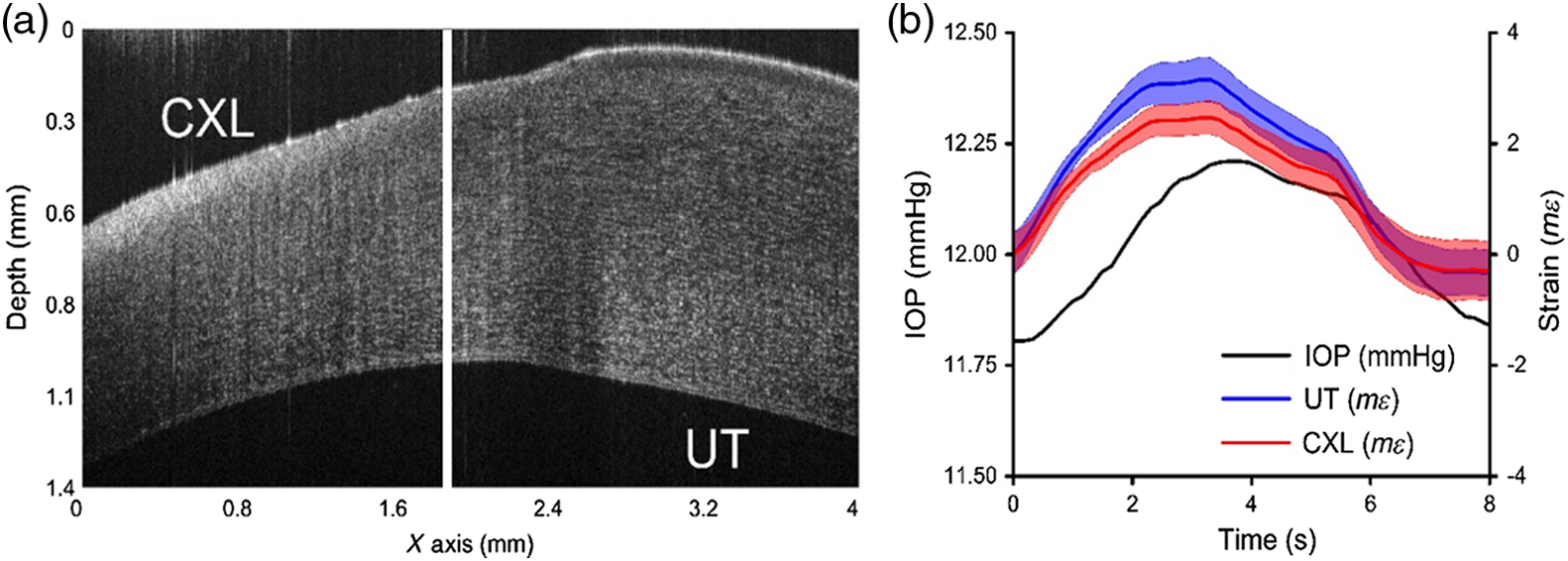
(a) Structural OCT image of a partially CXL porcine cornea, where the left side was CXL treated and the right side was untreated (UT). (b) Cumulative strain for the UT and CXL regions of the cornea are shown with IOP over time.

As a proof of concept, a partially CXL cornea was tested using the proposed Hb-OCE method. The structural OCT image shown in Fig. 4(a) illustrates that the clear change in thickness and optical scattering between each side indicates successful crosslinking. Figure 4(b) shows that the strain was different between the two regions of the cornea, suggesting a difference in stiffness.

**Fig. 5:**
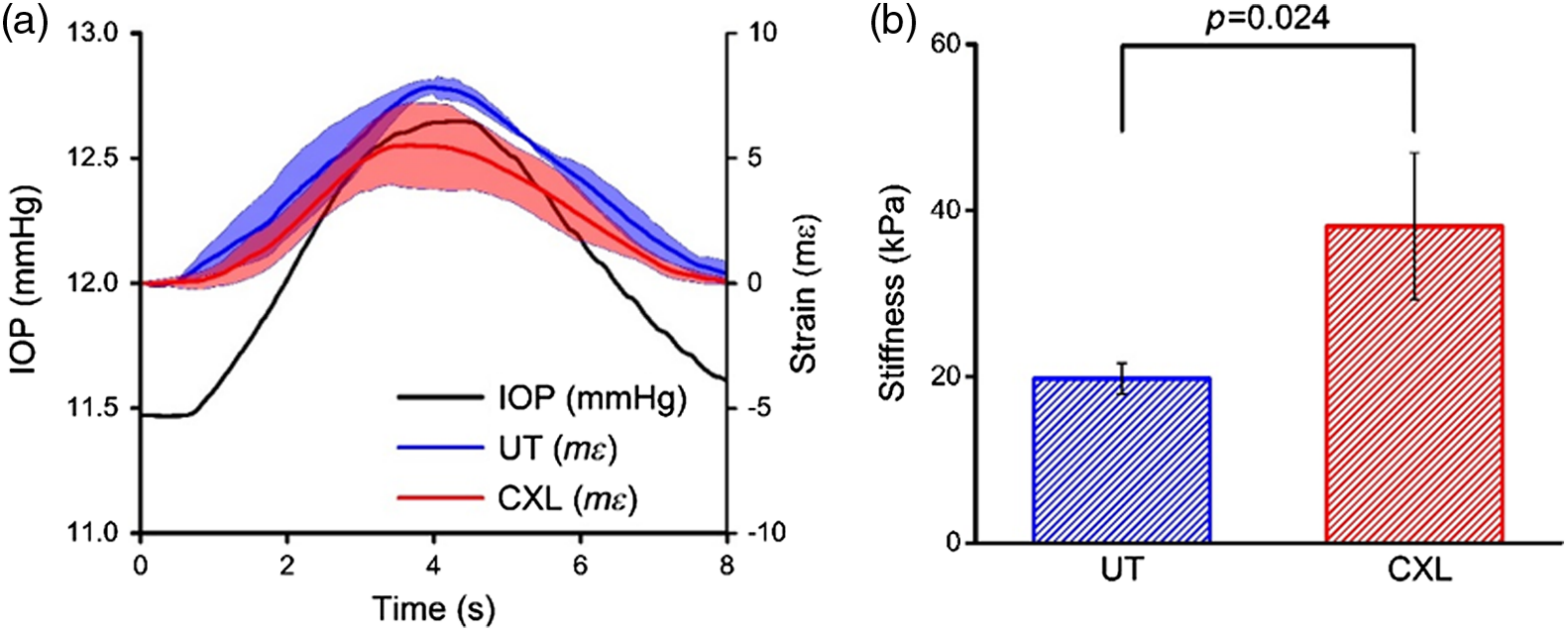
(a) Cumulative strain (mean±standard deviation of all samples, N=3 for each group). (b) Summary of sample stiffness (mean standard deviation of all samples) for untreated and crosslinked corneas. Results from a two-sample t-test are shown.

Untreated and CXL corneas for all three pairs are compared in Fig. 5. Cumulative strains in all corneas are shown; note that there is a distinct difference in cumulative strain between the two tissue types in all pairs. The maximum detected strain was normalized to the corresponding change in IOP to obtain stiffness with the following relationship:

stiffness=ΔIOP/strain.

Note that this stiffness parameter approximates the form of Young’s modulus by assuming the stress on the sample is solely the IOP. The stiffness in the untreated cornea was 19.8±1.8  kPa, and the stiffness in the crosslinked corneas was 38.1±8.8  kPa, resulting in a 93% increase in corneal stiffness after CXL. The difference in measured stiffness between untreated and CXL corneas was significant (p=0.024) based on a two-sample t-test.

## 4 Discussion and Conclusion

The results of this work suggest that the IOP fluctuations similar to that produced by the ocular pulse can be used as a natural excitation source for OCE measurements and can distinguish corneas of different stiffness. OCE with an external excitation source has been used to assess the mechanical properties of the cornea *in vivo* using dynamic wave propagation methods as well as quasistatic techniques.^19^^,^^21^^,^^42^ However, these techniques have a variety of limitations in acquisition and analysis, such as patient discomfort, nonlinear mechanical behavior, or lengthy imaging times. Since the technique presented in this work does not require complex wave analysis or additional tools to determine tissue mechanical properties, we can assess the mechanical properties of the cornea using only a phase-sensitive OCT system, a ubiquitous tool in ophthalmology and optometry clinics. Furthermore, since displacement is measured at individual pixels, we can potentially maximize displacement resolution to approach the OCT system.

Currently, Hb-OCE is primarily limited to relative mechanical contrast through strain rather than quantitative measurements of tissue elastic modulus. Similar elastography techniques utilize metrics based on IOP, but they are not standard measurements of elasticity.^25^^,^^27^ Quantification of elasticity has been performed here using a similar method in which IOP was treated as the stress on the cornea. Based on this metric, we calculated a 93% increase in the stiffness of cornea after crosslinking, which is within the range of results obtained in other works.^43^ Other methods of corneal elasticity assessment based on external excitation techniques have resulted in direct measurements of Young’s modulus, which is a useful metric for monitoring corneal stiffness over time or comparing different samples.^16^^,^^44^ Future work will focus on developing more robust models that can more accurately link the behavior of the cornea during pulsation to quantitative material parameters such as Young’s modulus.^45^ This would be particularly useful for quantitatively assessing the stiffness of the custom crosslinked cornea, where changes in geometry and hydration state are known to affect the measured mechanical properties of tissue.^46^^,^^47^

It should be noted that the pulse period, as well as the pressure amplitude used here, do not precisely represent natural physiological conditions. Pressure fluctuations due to IOP are between 1 and 5 mmHg on average, and the normal heart rate for humans is ∼0.75 to 1.67 Hz.^25^^,^^28^ Moreover, the rapid movement of the whole body and motion in the cornea due to the corneal pulse and saccades present a concern for Hb-OCE imaging *in vivo*. However, motion compensation issues can be mitigated with faster imaging speeds and established motion correction techniques.^48^^,^^49^ Our preliminary results here suggest that using IOP fluctuations to assess corneal stiffness is still feasible for *in vivo* assessment of corneal elasticity, which is the next step of our work.

There is well-documented evidence suggesting that IOP correlates with stiffness.^50^^–^^54^ One limitation of this work is that IOP effects on the cornea have not been investigated. Changes in the baseline IOP, as well as the accuracy of IOP measurements, may affect the Hb-OCE measured stiffness. It should be noted that, with this type of assessment, the effects of IOP may be distinguishable from the true stiffness of the corneal tissue.^30^ Investigating the effects of IOP and corneal stiffness using Hb-OCE will be another area of future work.

Another potential application of the described Hb-OCE method for *in vivo* applications is a potential to detect different pathophysiological conditions of the blood and the vessels. For example, diabetes, glaucoma, and other conditions are known to cause changes in ocular blood vessels and flow.^55^^,^^56^ This may in turn affect the ocular pulse and pulse shape and subsequent corneal displacement. This potential correlation deserves further investigation.

In summary, we have demonstrated that the heartbeat OCE can measure differences in corneal biomechanical properties with a truly passive and noncontact method. We demonstrated that there is a distinct difference in displacement and strain between untreated and CXL corneas imaged during a sinusoidal IOP pulsation. These results suggest that our method of Hb-OCE utilizing the natural pulsation in the cornea due to the heartbeat may be useful for assessing the stiffness of the cornea in the clinic, using only an OCT system and no additional instruments.
